# Improving Bloom Filter Performance on Sequence Data Using *k*-mer Bloom Filters

**DOI:** 10.1089/cmb.2016.0155

**Published:** 2017-06-01

**Authors:** David Pellow, Darya Filippova, Carl Kingsford

**Affiliations:** ^1^ The Blavatnik School of Computer Science, Tel Aviv University, Tel Aviv, Israel.; ^2^ Roche Sequencing Solutions, Pleasanton, California.; ^3^ Computational Biology Department, School of Computer Science, Carnegie Mellon University, Pittsburgh, Pennsylvania.

**Keywords:** efficient data structures, genomics, string algorithms, Bloom fitters, *k*-mers.

## Abstract

**Using a sequence's *k*-mer content rather than the full sequence directly has enabled significant performance improvements in several sequencing applications, such as metagenomic species identification, estimation of transcript abundances, and alignment-free comparison of sequencing data. As *k*-mer sets often reach hundreds of millions of elements, traditional data structures are often impractical for *k*-mer set storage, and Bloom filters (BFs) and their variants are used instead. BFs reduce the memory footprint required to store millions of *k*-mers while allowing for fast set containment queries, at the cost of a low false positive rate (FPR). We show that, because *k*-mers are derived from sequencing reads, the information about *k*-mer overlap in the original sequence can be used to reduce the FPR up to 30 × with little or no additional memory and with set containment queries that are only 1.3 – 1.6 times slower. Alternatively, we can leverage *k*-mer overlap information to store *k*-mer sets in about half the space while maintaining the original FPR. We consider several variants of such *k*-mer Bloom filters (*k*BFs), derive theoretical upper bounds for their FPR, and discuss their range of applications and limitations.**

## 1. Introduction

Many algorithms central to biological sequence analysis rely, at their core, on *k*-mers—short substrings of equal length derived from the sequencing reads. For example, sequence assembly algorithms use *k*-mers as nodes in the de Bruijn graph (Zerbino and Birney, 2008; Pell et al., 2012), metagenomic sample diversity can be quantified by comparing the sample's *k*-mer content against a database (Wood and Salzberg, 2014), *k*-mer content derived from RNA-seq reads can inform gene expression estimation procedures (Patro et al., 2014), and *k*-mer-based algorithms can dramatically improve compression of sequence (Rozov et al., 2014; Benoit et al., 2015) and quality values (Yu et al., 2014).

A single sequencing data set could generate hundreds of millions of *k*-mers making *k*-mer storage a challenging problem. Bloom filters (BFs) (Bloom, 1970) are often used to store sets of *k*-mers because they require much less space than hash tables or vectors to represent the same *k*-mer set while retaining the ability to quickly test for the presence of a specific *k*-mer at the cost of a low false positive rate (FPR) (Malde and O'Sullivan, 2009; Shi et al., 2009; Stranneheim et al., 2010; Marçais and Kingsford, 2011; Pell et al., 2012; Chikhi and Rizk, 2013; Heo et al., 2014; Rozov et al., 2014; Salikhov et al., 2014; Song et al., 2014; Holley et al., 2015; Solomon and Kingsford, 2016). For example, BFs allow for efficient *k*-mer counting (Marçais and Kingsford, 2011), can be used to represent de Buijn graphs in considerably less space (Pell et al., 2012), and can enable novel applications like inexact sequence search over very large collections of reads (Solomon and Kingsford, 2016).

The small size of BFs has allowed algorithms to efficiently process large amounts of sequencing data. However, smaller BF sizes have to be traded off against higher FPRs: a smaller BF will incorrectly report the presence of a *k*-mer more often. Sequencing errors and natural variation noticeably increase *k*-mer set sizes, with recent long read data driving *k*-mer set sizes even higher because of these data's lower overall quality profiles. To support large *k*-mer sets, researchers can increase the BF size, choose a more costly function to compute set containment, or attempt to reduce the FPR through other means.

To eliminate the effects of BF's false positives when representing a probabilistic de Bruijn graph (Pell et al., 2012)—where two adjacent *k*-mers represent an implicit graph edge—one can precompute the false edges in the graph and store them separately (Chikhi and Rizk, 2013). The results of querying the BF for a *k*-mer are modified such that a positive answer is returned only if the *k*-mer is not in the critical false positive set. The size of the set of critical false positives is estimated to be $6N \ f$, where *N* is the number of nodes in the graph (and the number of *k*-mers inserted into the BF) and *f* is the FPR of the BF (Salikhov et al., 2014). Cascading BFs lower the FPR by storing a series of smaller nested BFs that represent subsets of critical *k*-mers (Salikhov et al., 2014).

Although specific BF applications achieved improved false positive performance by using additional data structures, these applications assume the FPR of general-purpose BFs derived in the article that presented them originally (Bloom, 1970). This FPR is calculated based on the assumption that elements inserted into the BF are independent. In biological sequencing applications that store *k*-mers, the elements are not independent: if all *k*-mers of a sequence are stored, then there is a $k - 1$ character overlap between adjacent *k*-mers. The information about the presence of the *k*-mer's neighbors can be used to infer that the *k*-mer itself is part of the set—without the use of additional storage.

We use *k*-mer nonindependence to develop *k-mer Bloom filters* (*k*BFs) with provably lower FPRs. We first consider a *k*BF variant in which we are able to achieve more than threefold decrease in FPR with no increase in required storage and only a modest delay in set containment queries (1.2–1.3 × slower when compared with classic BF). We then consider a *k*BF with a stricter set containment criterion that results in more than 30-fold decrease in FPR with a modest increase in required storage and up to 1.9 × delay in set containment queries.

As the existence of *k*-mers in the BF can be inferred from the presence of neighboring *k*-mers, we can also drop certain *k*-mers entirely, sparsifying the BF input set. We implement sparsifying *k*BFs and achieve *k*-mer sets that are 51–60% the size of the original set with a slightly lower FPR at the cost of slower set containment queries.

The space and speed requirements vary between different *k*BF variants, allowing for a multitude of applications. In memory-critical algorithms, such as sequence assembly (Pell et al., 2012) and search (Solomon and Kingsford, 2016), sparse *k*BF can lower memory requirements, allowing to process larger read collections. Applications relying on *k*-mers for error correction (Song et al., 2014) or classification (Wood and Salzberg, 2014) may benefit from using *k*BF with guaranteed lower FPRs to confidently identify sequencing errors and to distinguish between related organisms in the same clade.

## 2. Reducing False Positive Rate Using Neighboring *k*-MERS

**FIG. 1.**
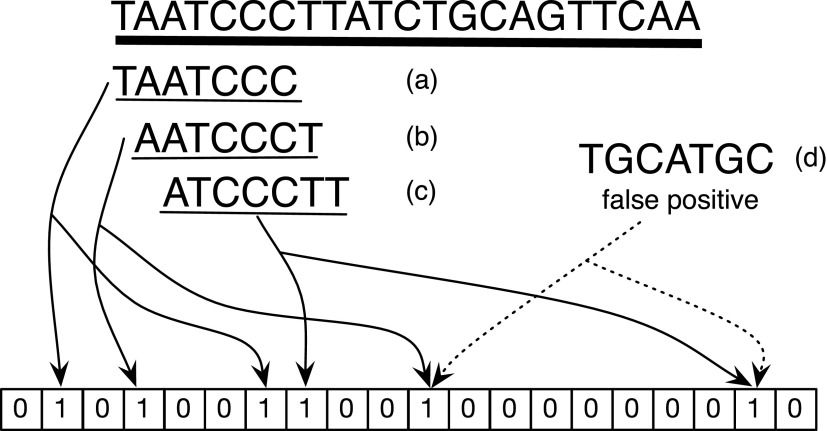
The *k*-mers from a sequence are stored in a Bloom filter. False positives could occur when the bits corresponding to a random *k*-mer not in the sequence are set because of other *k*-mers that are in the Bloom filter. The true *k*-mers from the sequence all share sequence overlaps with other true *k*-mers from the sequence. We show how this overlap can be used to reduce the false positive rate and sparsify the set of *k*-mers stored in the *k*BF. kBFs, *k*-mer Bloom filters.

When testing a BF for the presence of the query *k*-mer *q*, for example, AATCCCT (Fig. 1), the BF will return a positive answer—which could be a true or a false positive. However, if we query for the presence of neighboring *k*-mers xAATCCC *k*-mers (where *x* is one of $\{  A , C , G , T \}$) and receive at least one positive answer, we could be more confident that AATCCCT was indeed present in the BF. There is a nonzero chance that *q* is a false positive and its neighbor is itself a true positive; however, this is less likely than the chances of *q* being a false positive and thus lowers the FPR. We formally introduce the *k*BF and derive the probabilities of such false positive events hereunder.

### 2.1. One-sided *k*-mer Bloom filter

We define a *k*BF that only checks for the presence of a single overlapping neighbor when answering a set containment query as a *one-sided kBF* (1-*k*BF). Each *k*-mer *q* observed in the sequence or collection of sequencing reads is inserted into a BF *B* independently in the standard way. To test for *q*'s membership in a 1-*k*BF, the BF *B* is first queried for *q*. If the query is successful, then *q* is either in the true set of *k*-mers *U* or is a false positive. If $q \in U$ and all *k*-mers in *U* were added to the BF, then the set containment query for *q*'s neighbor should return “true.” We generate all eight potential left and right neighbors for *q* and test whether *B* returns true for any of them (Algorithm 1). Under the assumption that every read or sequence is longer than *k*, every *k*-mer will have at least one neighbor in the right or left direction.

**Algorithm 1:** One-sided *k*BF *contains* functions

1: **function** One-sided_kBF_contains(*query*)
2: **if** BF. contains(*query*) **then**
3: **return**contains_set(NEIGHBOR_SET(*query*))
4: **return false**
5: **function**contains_set(*set*)
6: **for***kmer* ∈ *set***do**
7: **if** BF. contains(*kmer*) **then return true**
8: **return false**

### 2.2. Theoretical false positive rate for a one-sided *k*-mer Bloom filter

We show that the theoretical upper bound for the FPR of a 1-*k*BF is lower than that for the classic BF (Bloom, 1970). Suppose we inserted *n* unique *k*-mers into a BF of length *m* using *h* hash functions. Then the expected proportion of bits that are still 0 is $E = { \left( {1 - 1 / m} \right) ^{hn}}$ and the actual proportion of zeros, *p*, is concentrated around this mean with high probability (Broder and Mitzenmacher, 2004). The FPR *f* is

\begin{align*}
f = ( 1 - p{ ) ^h} \approx { ( 1 - E ) ^h} = { \left( {1 - {{ \left( {1 - 1 / m} \right) }^{hn}}} \right) ^h}. \tag{1}
\end{align*}

Let $q \prime$ be *q*'s neighbor that overlaps *q* by $k - 1$ characters on either the left or the right side, and let *t_k_* be a probability that a random *k*-mer is a true positive (i.e., present in the set *U*). We assume further that the events “*q* is a false positive” and “$q \prime$ is a false positive” are independent because the false positives result from bits being set by uniform random hashes of other *k*-mers inserted into the BF. Then the chance that we get a false positive when testing for the presence of a random *k*-mer *q* and one of its eight neighbors $q \prime$ is

\begin{align*}
f {^{\prime}} = f \cdot { \rm Pr} ( { \rm BF \ returns } \
{^{\prime\prime}} {\rm True}{^{\prime\prime}  \,\rm for \ at \
least \ one \ of \ the \ adjacent} \ {\it k} {-} { \rm mers}
)\tag{2}
\end{align*}

\begin{align*}
 = f \cdot \left( 1 - { \rm Pr} ( { \rm BF \ returns \ } {^{\prime\prime}} {\rm
False}{^{\prime\prime} \,\rm  \ for \ every \ adjacent} \ {\it k}
{-} { \rm mer} ) \right) \tag{3}
\end{align*}

\begin{align*}
 = f \cdot \left( 1 - { \rm Pr} ( { \rm BF \ returns \
}{^{\prime\prime}}{\rm False}{^{\prime\prime} \,\rm  \ for \ an \
adjacent} \ {\it k} {-} { \rm mer} ) ^8 \right) \tag{4}
\end{align*}

\begin{align*}
 = f \cdot \left( 1 - ( 1 - { \rm Pr} ({\rm BF \ returns \ }{^{\prime\prime}}{\rm True}{^{\prime\prime} \,\rm \ for \ an \
adjacent} \ {\it k}{-} { \rm mer} ) ) ^8 \right) \tag{5}
\end{align*}

\begin{align*}
 = f \cdot \left( {1 - {{ ( 1 - ( f + {t_k} ) ) }^8}} \right) . \tag{6}
\end{align*}

Assuming that *k*-mers are uniformly distributed, we can estimate *t_k_* as the chance of drawing a *k*-mer from the set *U* giving the set of all possible *k*-mers of length *k*, or ${t_k} = \vert U{ \vert / 4^k}$. For reasonably large values of $k \ge 20$, *t_k_* will be much smaller than *f*, allowing us to estimate an upper bound on $f {^{\prime}}$:

\begin{align*}
f {^{\prime}} < f \cdot \left( {1 - {{ ( 1 - 2f ) }^8}} \right) , \tag{7}
\end{align*}

quantifying how much lower $f {^{\prime}}$ is than the FPR *f* for the classic BF.

### 2.3. Two-sided *k*-mer Bloom filter

The FPR could be lowered even more by requiring that there is a neighboring *k*-mer extending the query *k*-mer in both directions to return a positive result. This is a two-sided *k*-mer Bloom filter (2-*k*BF). However, this requires dealing with *k*-mers at the boundary of the input string. In Figure 1, it can be seen that the first *k*-mer (TAATCCC) only has a right neighbor and no left neighboring *k*-mers. We call this an *edge k-mer,* which must be handled specially, otherwise the 2-*k*BF would return a false negative.

To avoid this, 2-*k*BF maintains a separate list that contains these edge *k*-mers. We augment the BF with a hash table EDGE_k-mer_set that stores the first and last *k*-mers of every sequence to handle edge cases. When constructing the *k*BF from a set of sequence reads, the first and last *k*-mers of each read are stored. As reads can overlap, many of the read edge *k*-mers will not be true edges of the sequence. After all the reads have been inserted into the BF, each of the stored *k*-mers is checked to see whether it is an edge *k*-mer of the sequence, and if it is, then it is saved in the final table of edge *k*-mers. The only *k*-mers that will be stored in the final edge table are those at the beginning and end of the sequence, or those adjacent to regions of zero coverage. Pseudocode for querying a 2-*k*BF is given in Algorithm 2.

**Algorithm 2:** Two-sided *k*BF *contains* function

1: **function**two-sided_kBF_contains(*query*)
2: **if** BF.contains(*query*) **then**
3: Contains_left $\leftarrow$contains_set(LEFT_NEIGHBOR_SET(*query*))
4: Contains_right $\leftarrow$contains_set(RIGHT_NEIGHBOR_SET(*query*))
5: **if** Contains_right = = **true and** Contains_left = = **true then**
6: **return true**
7: **if** Contains_right = = **true or** Contains_left = = **true then**
8: **if** EDGE_*k*-mer_SET.contains(*query*) **then**
9: **return true**
10: **return** FALSE

### 2.4. Theoretical false positive rate for a two-sided *k*-mer Bloom filter

Ignoring edge *k*-mers for simplicity and following the same assumptions and derivation as in Section 2.2., the FPR for 2-*k*BF, $f {^{\prime}}$, is

\begin{align*}
\begin{split}
f {^{\prime}} = f \cdot & {\rm Pr} ( { \rm BF \ returns } \
{^{\prime\prime}} {\rm True}{^{\prime\prime}  \,\rm \ for \ at \
least \ one \ of \ the \ left\ adjacent} \ {\it k} {-} { \rm
mers})\cdot \\ & \; {\rm Pr} ( { \rm BF \ returns } \
{^{\prime\prime}} {\rm True}{^{\prime\prime}  \,\rm \ for \ at \
least \ one \ of \ the \ left\ adjacent} \ {\it k} {-} { \rm
mers)\cdot } \end{split} \tag{8}
\end{align*}

This leads to

\begin{align*}
f {^{\prime}} = f \cdot { \left( {1 - {{ ( 1 - ( f + {t_k} ) ) }^4}} \right) ^2}. \tag{9}
\end{align*}

An upper bound for this expression can be estimated as

\begin{align*}
f {^{\prime}} < f \cdot { \left( {1 - {{ ( 1 - 2f ) }^4}} \right) ^2}. \tag{10}
\end{align*}

## 3. Using Sequence Overlaps to Sparsify *k*-Mer Sets

We can use the assumption that the set of *k*-mers to be stored, *U*, contains *k*-mers derived from an underlying string *T* to reduce the number of *k*-mers that must be stored in *B* without compromising the FPR. If we want to store a set *U*, we can choose a subset $K \subseteq U$ that will be stored in *B*. The idea is that every *k*-mer $u \in U$ will have some neighbors that precede it and some that follow it in the string *T*.

Let ${L_u} \subset U$ be a set of *k*-mers that occur before *u* in *T*, and let ${R_u} \subset U$ be a set of *k*-mers that occur after *u* in *T*. If we can guarantee that there is at least one *k*-mer of *L_u_* and at least one *k*-mer of *R_u_* that are close to *u* stored in *B*, then we can infer the presence of *u* from the presence of $v \in {L_u}$ and $w \in {R_u}$ without having to store $u \in B$. By reducing the *k*-mers that must be kept in *B*, we can maintain the set *U* using a smaller filter *B*. For example, in Figure 1 the *k*-mer AATCCCT is preceded by TAATCCC and followed by ATCCCTT. If these two *k*-mers are stored in *B* then the presence of the middle *k*-mer AATCCCT in the sequence can be inferred without having to store it in the BF.

More formally, define ${P_{vu}}$ to be the set of positions of *k*-mer *v* occurring before *u* in *T*, and let ${A_{uw}}$ be the set of positions of *k*-mer *w* occurring after *u* in *T*. We then define, for $v \in {L_u}$ and $w \in {R_u}$, the set of all distances between occurrences of these *k*-mers that span *u*:

\begin{align*}
{S_u} ( v , w ) = \{  {i_w} - {i_v} \vert {i_v} \in {P_{vu}},{i_w} \in {A_{uw}} \}  .
\end{align*}

For some *skip length s*, if we can guarantee that $\min {S_u} ( v , w ) \le s$ for some $v , w \in K$, then we can infer the presence of *u* without storing it in the BF by searching for neighboring *k*-mers that satisfy $\min {S_u} ( v , w ) \le s$. This leads to the following *k*-mer sparsification problem.

**Problem 1 (Relaxed** ***k*-mer sparsification)** *Given a set of k-mers U, find a small subset* $K \subset U$ *such that for all* $u \in U$*, either* $u \in K$ *or there is a k-mer* $v \in K \cap {L_u}$ *and* $w \in K \cap {R_u}$ *with* $\min {S_u} ( v , w ) \le s$.

We call this problem the *relaxed k*-mer sparsification problem. When we require exactly *s* skipped *k*-mers between those *k*-mers chosen for *K*, we have the *strict k*-mer sparsification problem.

**Problem 2 (Strict** ***k*-mer sparsification)** *Given a set of k-mers U, find a small subset* $K \subset U$ *such that for all* $u \in U$*, either* $u \in K$ *or there is a k-mer* $v \in K \cap {L_u}$ *and* $w \in K \cap {R_u}$ *with* $s \in {S_u} ( v , w )$.

In practice, *s* should be a small integer because the FPR and running time are expected to increase quickly as *s* increases.

These sparsification problems would be easy if we could observe *T*—a solution would be to select every *s*th *k*-mer (Approach 3 hereunder). However, we assume that we see only short reads from *T* and must select *K* as best as possible. Hereunder, we propose three solutions to the *k*-mer sparsification problem that are appropriate in different settings.

**Approach 1: Best index match per read sparsification (*k*-mers come from reads; arbitrary** *s***).** *K* will be built greedily by choosing *k*-mers from each read. Given a read *r*, we choose every *s*th *k*-mer starting from a particular index *i_r_*, choosing *i_r_* such that the set of *k*-mers *K_r_* chosen for this read has the largest intersection with the set of *k*-mers *K* chosen so far.

**Approach 2: Sparsification through approximate hitting set (*k*-mers come from reads;** ***s* = *1*).** When $s = 1$, the relaxed *k*-mer set sparsification problem can also be formulated as a minimal hitting set problem: For each *k*-mer $k \in U$, create a set *L_k_* that includes *k* and every *k*-mer that immediately preceded it in some read, and a set *R_k_* that includes *k* and every *k*-mer that immediately followed it in some read. Let $L = \{  {L_k}:k \in U \}$ and $R = \{  {R_k}:k \in U \}$. A solution to the minimal hitting problem chooses a minimum size set *K* such that at least one *k*-mer from *K* is in every set in *R* and *L*: $\forall N \in \{  R \cup L \}  \ \exists k \in K \ { \rm{s}}{ \rm{.t}}{ \rm{.}} \ k \in N$ and $\vert K \vert$ is minimized. We use a greedy approximation for the hitting set problem to choose *K*, the sparse set of *k*-mers. In each step of the greedy approximation, we add the *k*-mer that hits the most sets in $L \cup R$ to *K*.

**Approach 3: Single sequence sparsification (k-mers come from a known sequence** *T***; arbitrary** ***s*).** In the special case in which input sequences are nonoverlapping (e.g., a genome or exome) rather than multiple overlapping sequences (e.g., the results of a sequencing experiment), we solve the strict *k*-mer sparsification problem by taking each *k*-mer starting from the beginning of the sequence and then skipping *s k*-mers. This is a simple and fast way to choose the sparse set *K*, but restricted only to this special case, and will not work for sparsifying the *k*-mers from a set of reads generated in a sequencing experiment. It is useful, for example, if the input sequences are a reference genome that will be queried against.

Once *K* has been chosen, a sparse *k*BF can be queried for *k*-mers from *U* using Algorithm 3. Two different query functions are given: relaxed-contains for when *K* satisfies the conditions of Problem 1 and strict-contains for when *K* satisfies the conditions of Problem 2. We call two *k*-mers with *s* skipped *k*-mers between them *s*-distant neighbors. The helper function contains_neighbors determines whether *k*-mers neighboring the query *k*-mer at specified distances to the left and right are present and decide_present determines whether the query is present depending on whether it has neighboring *k*-mers or is an edge. The sparse *k*BF also maintains a set of edge *k*-mers that is queried when a *k*-mer has neighbors in one direction but not the other.

**Algorithm 3:** Sparse *k*BF *contains* functions

1: **function**decide_present($$query , Contains \_left , Contains \_right$$)
2: **if** Contains_right = = **true and** Contains_left = = **true then**
3: **return true**
4: **If** Contains_right = = **true or** Contains_left = = **true then**
5: **if** EDGE_*k*-mer_SET.contains(*query*) **then**
6: **return true**
7: **return false**
8: **function**strict-contains_neighbors($query , left \_dist , right \_dist$)
9: Contains_left $$\leftarrow$$contains_set(s_distant_left_neighbor_set($$query , left \_dist$$))
10: Contains_right $$\leftarrow$$contains_set(s_distant_right_neighbor_set($$query , right \_dist$$))
11: **return**decide_present(*query*, $Contains \_left$, $Contains \_right$)
12: **function**relaxed-contains_neighbors($query , l \_dist , r \_dist$)
13: Contains_left $$\leftarrow$$contains_set($$\mathop \bigcup \limits_{i \le l \_dist}$$s_distant_left_neighbor_set($$query , i$$))
14: Contains_right $$\leftarrow$$contains_set($$\mathop \bigcup\limits_{i \le r \_dist}$$s_distant_right_neighbor_set($$query , i$$))
15: **return**decide_present(*query*, $Contains \_left$, $Contains \_right$)
16: **function**strict-contains($query , s$)
17: **if** BF.contains(*query*) **then**
18: **if**strict-contains_neighbors($query , s,s$) **then**
19: **return true**
20: **for**$i \leftarrow 0$**to**$s - 1$**do**
21: **if**strict-contains_neighbors($query , i , s - ( i + 1 )$) **then**
22: **return true**
23: **return false**
24: **function**relaxed-contains($query , s$)
25: **if** BF.contains(*query*) **then**
26: **if**relaxed-contains_neighbors($query , s,s$) **then**
27: **return true**
28: **else**
29: **for**$$i \leftarrow 0{ \kern 1pt} \ \to \ \ s - 1$$**do**
30: **if**relaxed-contains_neighbors($query , i , s - ( i + 1 )$) **then**
31: **return true**
32: **return false**

## 4. Results and Discussion

We test the performance of the proposed *k*BFs on a variety of sequencing experiments and compare with classic BFs. For each test, we store the *k*-mers from the input file in the *k*BF and create a query set by mutating *k*-mers from the input. We test on multiple species and types of experiments that could typically be used in applications that require BFs over a range of input file sizes. The different data sets are summarized in Table 1.

**Table 1.** Read Sets on Which *k*-mer Bloom Filter Variants Were Tested

*Accession*	*Type*	*Read count*	*Read length*
ERR233214_1	WGS of *Pseudomonas aeruginosa*	7,571,879	92
SRR1031159_1	Metagenomic, WGS	674,989	101
SRR514250_1	Metagenomic, WGS	44,758,957	100
SRR553460	Human RNA-seq	66,396,200	49
chr15	Human chromosome (hg19)	1	81Mbp

Only reads without “N” bases were included.

kBFs, *k*-mer Bloom filters; WGS, whole-genome sequencing.

For all tests, we used a *k*-mer length of $k = 20$ and two hash functions in the underlying BF. This choice of *k* is long enough that only a fraction of all possible *k*-mers are present in reasonably large data sets and shorter than all read lengths and is representative of *k*-mer lengths used in practice. The BF length is 10 times the number of *k*-mers inserted into it for each of the input files. For 1-*k*BF and 2-*k*BF, the underlying BF will be exactly the same as the classic BF it is compared to. For sparse *k*BF, the smaller sparse *k*-mer set is stored, so the underlying BF is smaller. The sparse *k*BFs use a skip length of $s = 1$. The implementations of the *k*BF variants described wrap around the basic BF implementation from libbf (http://mavam.github.io/libbf), which is used for the classic BF.

To create a query *k*-mer set for testing, we randomly select (uniformly, with replacement) 1 million *k*-mers from the input file and mutate one random base. This creates a set of *k*-mers that are close to the real set, and will, therefore, have realistic nucleotide sequences while still providing many negative queries to test the FPR. For one experiment (SRR1031159), we also query with 1 million true queries (not mutated) to determine the worst-case impact on query time.

### 4.1. One-sided and two-sided *k*-mer Bloom filter performance

**FIG. 2.**
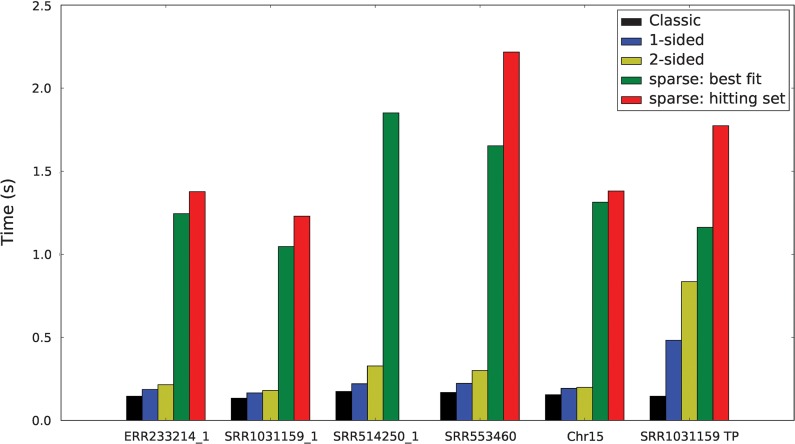
Query times. Comparison of the time to query 1 million *k*-mers in classic BF and the different *k*BF implementations (average of 10 runs).

The 1-*k*BF and 2-*k*BF implementations achieve substantially better FPRs than the classic BF (Table 2) at the cost of some query time overhead (Fig. 2). For the 1 million mutated queries, only about one-quarter of the queries are true positives, and 1-*k*BF and 2-*k*BF take 1.3 and 1.6 times as long to perform the queries, respectively. In the worst case, when all of the queries are true positives (SRR1031159 TP), 1-*k*BF and 2-*k*BF are 3.3 and 5.8 times slower, respectively, whereas the speed of the classic BF does not change.

**Table 2.** False Positive Rates

				*Sparse*	
*Accession*	*Classic*	*1-kBF*	*2-kBF*	*Best match*	*hitting set*
ERR233214_1	0.0329	0.0104	0.0009	0.0284	0.0311
SRR1031159_1	0.0329	0.0104	0.0009	0.0279	0.0306
SRR514250_1	0.0329	0.0106	0.0010	0.0290	—
SRR553460	0.0329	0.0104	0.0009	0.0285	0.0314
Chr15	0.0328	0.0104	0.0009	0.0284	0.0309
Theoretical FPR	0.0328	<0.0138	<0.0019	—	—

Comparison of FPRs for classic Bloom filters and the different *k*BF implementations. The theoretical FPRs are also shown in the last row [calculated according to Eqs. (1), (7), and (10)]. Hitting set sparsification uses the relaxed contains function, whereas best match uses the strict contains function. The sparse hitting set results for SRR514250_1 are missing because the method never completed on this data set.

FPR, false positive rate.

1-*k*BF has an FPR less than one-third of the classic BF FPR at a cost of an extra one-third the query time. Query times are extremely low, and this extra cost totals less than half a second to perform 1 million queries.

The 2-*k*BF requires a special data structure that stores the set of edge *k*-mers that may not be found because there is no adjacent *k*-mer on one side. The total number of *k*-mers and the number of *k*-mers in the edge set of each file are compared in Table 3. There is also extra memory and speed overhead during the 2-*k*BF creation: as the sequence file is read in and split into *k*-mers, a set of *k*-mers at the edges of all reads (which could potentially be sequence edges that need to be stored separately) is maintained. After *k*-mers are inserted into the BF, the edges are checked, and only the true sequence edges, which do not have neighboring *k*-mers on both sides, are stored. We do not optimize the *k*BF implementations for the one-time cost of creating the *k*-mer set and populating the BF, but note that the potential edge set could be pruned on the fly, keeping it smaller than the maximum size achieved here. We report the number of potential edge *k*-mers stored in the edge set and the amount of extra time to check the edges in Table 3. In all tested cases, the number of edge *k*-mers stored is a small fraction of the total number of *k*-mers, reaching at most 6% of the total. It is smallest when there are very few true sequence edges (in the single chromosome) and can be large if there are many reads with errors in the edge *k*-mers or many areas with zero coverage. If this overhead can be tolerated, applications could use 2-*k*BF to achieve significantly lower FPRs.

**Table 3.** Two-Sided *k*-mer Bloom Filter Overhead

*Accession*	*No. of k-mers*	*No. of edge k-mers*	*No. of potential edge k-mers*	*Initialization time (fold change)*
ERR233214_1	41,766,273	1,134,617	6,310,923	1.632 ×
SRR1031159_1	29,937,099	632,996	1,088,645	1.162 ×
SRR514250_1	442,498,904	6,656,205	53,063,633	1.813 ×
SRR553460	196,863,538	12,271,956	38,806,654	2.453 ×
Chr15	70,240,374	1	2	0.909 ×

The number of extra edge *k*-mers that are stored for two-sided *k*BF (2-*k*BF) is compared with the total number of *k*-mers. The one-time initialization overhead includes keeping track of all potential edge *k*-mers and extra time to query which are the true edge *k*-mers. The number of potential edge *k*-mers is compared with the number of true edge *k*-mers and with the total number of *k*-mers. The initialization time for 2-*k*BF is shown as a fold-change over populating the classic Bloom filter with the *k*-mer set (average of 10 runs).

2-*k*BF provides an FPR that is 30$\times$ smaller than classic BFs with a small query time penalty. 2-*k*BF also has a one-time cost of initialization to keep track of all potential edge *k*-mers and then determine the true edges. The extra time for this is only a small fraction of the total initialization cost. However, a large number of potential edge *k*-mers are stored during initialization. This number depends on the number of unique reads, and for the data sets with few long reads, that is, the single chromosome, there is very little overhead, whereas when there are many reads, the first and last *k*-mers of each read could be stored.

### 4.2. Sparse *k*-mer Bloom filter performance

The sparse *k*BF implementations achieve slightly better FPRs than the classic BFs while using a smaller filter. We report the FPRs for the best index match and hitting set implementations of sparse *k*BF in Table 2. We do not report specific results for single sequence sparsification (Approach 3) because we found in practice the results are the same as for best match sparsification in the cases in which it is relevant. When a sparse set of *k*-mers is used, sparse *k*BF is able to use the sequence overlap to recover a similar FPR for this smaller set of *k*-mers.

The sparsification performance of the different implementations is compared in Table 4. The sparsification methods perform well, with the best match achieving close to the ideal size of one half the number of *k*-mers. The hitting set sparsification method does not perform as well, choosing a *k*-mer set that is roughly 10% larger than the best match method.

**Table 4.** Number of *k*-mers Selected by Sparse *k*-mer Bloom Filter

*Accession*	*No. of k-mers classic*	*No. of k-mers best match*	*No. of k-mers hitting set*
ERR233214_1	41,766,273	21,783,670	23,635,764
SRR1031159_1	29,937,099	15,120,795	16,992,976
SRR514250_1	442,498,904	237,264,629	—
SRR553460	196,863,538	102,224,726	115,593,454
Chr15	70,240,374	36,064,290	39,152,979

Comparison of the number of *k*-mers in the sparsified *k*-mer set for the different implementation methods and for the classic Bloom filter.

Sparse *k*BF queries are significantly slower than classic BF queries. The speeds to perform 1 million queries for the classic BF and the different sparse *k*BF implementations are shown in Figure 2. The time overhead of querying neighboring *k*-mers is about 10 times that for a classic BF, but is still only around 1–2 seconds for 1 million queries. In memory-constrained applications, it could be worth paying this time penalty for smaller *k*-mer sets. The time overhead will grow exponentially as *s* is increased, but even very small *s* (such as $s = 1$ shown in our experiments) significantly reduces the size of the stored *k*-mer set. Similarly, as *s* increases, the FPR will increase, but as shown here, for small *s*, the FPR is comparable to the FPR of a classic BF.

The hitting set sparsification implementation has a very large memory footprint and takes a lot of time to choose the sparse *k*-mer set. For the largest file (SRR514250), the implementation uses up all available RAM and does not complete after running for several days. Table 5 compares the total time to split the input sequences into *k*-mers, choose the *k*-mer set, determine the edge *k*-mers, and populate the BF for the different sparsification implementations and 2-*k*BF. We compare with 2-*k*BF because it also has edge *k*-mers, making it the most similar nonsparse implementation to the sparse *k*BF implementations. The memory overhead of initialization (measured as the maximum resident set size of the process) is also compared in Table 5.

**Table 5.** Sparse *k*-mer Bloom Filter Overhead

	*Initialization memory overhead (GB)*			*Initialization time overhead (sec)*		
*Accession*	*2-kBF*	*Best match*	*Hitting set*	*2-kBF*	*Best match*	*Hitting set*
ERR233214_1	3.45	4.26	42.00	121.9901	192.7540	857.8472
SRR1031159_1	1.62	2.07	28.67	21.7156	21.5318	373.4421
SRR514250_1	29.54	38.41	—	1342.1470	1856.5640	—
SRR553460	17.85	22.55	198.18	699.4796	932.5057	6012.9880
Chr15	3.94	4.49	67.04	43.5708	25.7702	844.1708

Comparison of the one-time overhead for the initialization of the sparse *k*BF implementations. The one-time cost of splitting the sequences into *k*-mers, choosing the *k*-mer set, checking the edge *k*-mers, and inserting them into the Bloom filter is reported. The hitting set implementation for SRR514250_1 used up all available memory and did not complete running. Results are the averages over 10 runs.

The relaxed *contains* function, which must be used when *K* is selected using the hitting set formulation, needs to check more possible neighboring *k*-mers, making the hitting set sparsification queries slower than the other implementations. The hitting set implementation also does not do as good a job of sparsifying the original set of *k*-mers. Hitting set sparsification also takes orders of magnitude more memory and time than the other methods and the nonsparse *k*BF implementation.

In contrast to the hitting set sparsification, best match sparsification achieves close to one half of the original *k*-mer set with little extra overhead in initialization time or memory. The strict *contains* function for sparse *k*BF also has a better FPR than the relaxed version and takes less time to perform 1 million queries. In practice, there is little difference between the best match sparsification and single sequence sparsification, because they will both yield approximately the same *k*-mer set in a case in which single sequences are being sparsified. These results mean that best match sparsification is the simplest and best way to sparsify any set of sequences, without having to determine whether it is a special case of nonoverlapping sequences.

## 5. Conclusion

Together, the possibilities of drastically reducing the FPR or reducing the size of the BF have the potential to enable continued performance improvements in many applications that use BFs to store *k*-mers from sequences. Such performance improvements are necessary to allow biological sequence applications to continue to scale to larger and many more experiments. One direction for future work is improving the running time of the sparse variants, which, although still fast, incur a slowdown compared with the nonsparse versions. We expect that 1-*k*BF, 2-*k*BF, and sparse *k*BF data structures will be useful in genome assembly, sequence comparison, and sequence search applications, among other genomic analysis problems. See the first Reference for a reference implementation of kBF (Pellow et al., 2016).
